# Knowledge and attitudes of concussion and chronic traumatic encephalopathy amongst a sample of non-professional rugby players

**DOI:** 10.1007/s11845-025-04039-7

**Published:** 2025-08-12

**Authors:** Jack Moore, Conor Deasy

**Affiliations:** 1 University College Cork – School of Medicine, Cork, Ireland; 2 Cork University Hospital (CUH), Clinical Director of Emergency and Acute Care at CUH, Cork, Ireland

**Keywords:** Attitude, Chronic Traumatic Encephalopathy, Concussion, Knowledge, Sports

## Abstract

**Background:**

Sports related concussions are common in rugby. There is increasing awareness that repeated concussion may contribute to long-term neurological complications, including chronic traumatic encephalopathy (CTE).

**Objectives:**

To evaluate concussion knowledge (CK), concussion attitude (CA) and Chronic Traumatic Encephalopathy related knowledge (CTE-K) amongst non-professional rugby players’ in Ireland. To explore the impact gender, playing level, experience, and concussion history had on these outcomes. To identify interest in receiving further information and educational materials on CTE.

**Methods:**

Design: Cross sectional study.

**Setting & participants:**

A sample of non-professional rugby players (n = 529) registered with the Irish Rugby Football Union (IRFU) was recruited between July 2023 and December 2023 to complete either a paper based or online version of the Rosenbaum Concussion Knowledge and Attitude Survey-Student Version (RoCKAS-ST) to evaluate players concussion knowledge index (CKI) and concussion attitude index (CAI). An additional chronic traumatic encephalopathy knowledge index (CTE-KI) was created for this study.

**Results:**

The mean score of RoCKAS knowledge was 20.1 ± 2.4 (range 10–25) and attitudes 63.5 ± 7.2 (range 39–75). The mean CTE-KI was 4.90 ± .9 (range 0–6). Club players scored significantly higher than school players in all sections. 81.1% of players expressed interest in receiving further education relating to CTE.

**Conclusions:**

Non-professional rugby players are generally knowledgeable about concussion. However, some gaps in their knowledge relating to coma and the course of symptoms of concussion were identified. Unsafe attitudes, particularly among school players, need to be addressed. Non-professional rugby players are interested in further education about CTE.

**Supplementary Information:**

The online version contains supplementary material available at 10.1007/s11845-025-04039-7.

## Introduction

Concussions are a concern in contact sports, particularly in rugby. According to an Irish Rugby Injury Surveillance (IRIS) Report, concussion is the most common injury diagnosed in non-professional men’s and women’s rugby clubs in Ireland [1]. There is growing recognition in research that repeated concussions are a potential contributing factor in the development of long-term neurological complications, such as Chronic Traumatic Encephalopathy (CTE) [2], which has been detected in former rugby players [3, 4]. CTE is a progressive neurodegenerative disease characterized by cognitive decline, mood disturbances, and behavioural changes [2]. Currently, it can only be diagnosed post-mortem [5], which is based on a defined set neuropathologic criteria [6]. The corresponding clinical syndrome is known as traumatic encephalopathy syndrome (CTE) [7].

Several studies have assessed non-professional rugby players concussion knowledge (CK) and concussion attitude (CA), both in an Irish setting [8–10], and abroad [11–13]. Having safe knowledge and attitudes towards concussion is essential to protect the welfare and well-being of rugby players. However, available data indicates a high prevalence of players continuing to play in rugby despite being aware of their concussion, as well as a tendency to avoid seeking medical attention intentionally after a suspected concussion, particularly during high-pressure or ‘important’ games [9–12]. The Rosenbaum Concussion Knowledge and Attitudes Survey – Student Version (RoCKAS-ST) is a validated tool [14] that has been used to assess rugby players'CK and CA in some settings [11, 12], but not yet in Ireland. Previous studies using this tool in rugby have shown varied results [11, 12], suggesting that findings cannot be generalized across different populations and countries.

Within rugby in Ireland, return to play (RTP) guidelines are concussion protocols set by the Irish Rugby Football Union (IRFU) and World Rugby [15, 16], based on the latest medical advice from the 6th International Consensus Conference on Concussion in Sport [17]. These guidelines state that players should immediately leave the game if a concussion is suspected. The IRFU regularly updates its concussion educational materials [15], so it seems appropriate to assess the impact of these materials on players'concussion knowledge (CK) and attitudes (CA).

As no study has yet assessed players knowledge of CTE, it also seems appropriate to evaluate rugby players'awareness of the evidence linking CTE to head injuries [18–23], and their interest in receiving more educational materials on the topic. Understanding the knowledge and attitudes of non-professional rugby players towards concussion and CTE is essential for the development of targeted education and prevention strategies to promote player safety and minimize the risk of long-term neurological complications.

## Methods

### Study design & ethics

This cross-sectional study obtained ethical approval from the University College of Corks Social Research and Ethics Committee.

## Study sample and data extraction

The inclusion included being registered with the Irish Rugby Football Union (IRFU) as a player aged over 16 for either a club or a school during the 2023–24 season. Based on the most recent IRFU annual report [24] containing the number of registered club players (n = 20,000) and school players (n = 22,600), it was calculated that a minimum sample size of 381 non-professional rugby players was required using Cochran’s formula. This sample size ensured a 95% confidence level that the real value is within ± 5% of the surveyed value.

A complete list of clubs and schools is available on the IRFU [25] and Irish provincial rugby websites [26–29]. Contact was made via an invitation email to club and school representatives. The survey was initially administered online, but was later offered in both online and paper formats to improve response rates. Of the 32 clubs contacted, 17 agreed to participate. Ten clubs completed the survey online, while 7 opted for an in-person survey format. Four clubs declined to participate, and 11 did not respond. Among the 16 schools contacted, 5 agreed to participate. Three schools completed the survey online, and 2 opted for the in-person format. Two schools declined, and 9 did not respond.

Both the online and the in-person survey contained an introduction to the research, a plain language statement, and a consent form. Consenting to participate was needed to proceed with the questionnaire. There was no obligation based on the club’s decision rather than players’ to complete the survey. Paper-based surveys were completed before training sessions. Players completing the survey in person were spaced apart as much as possible, and silence was strongly encouraged whilst completing the survey. The online survey was conducted via Google Forms, with email or text links sent to the representatives of clubs and schools, who then shared them with the players.

## Measures

The following variables were explored to determine their impact on the outcome measures: age, gender, level of education, years of playing experience, position played, previous concussion education, number of suspected previous concussions, and the number of medically diagnosed concussions.

The initial part of the survey included sections on demographics (6 questions), previous concussion history (2 questions), and prior concussion education (2 questions). The remainder of the survey was composed of two parts: The RoCkAS and a CTE questionnaire.

The RoCKAS survey was used to investigate Concussion Knowledge (CK) and Concussion Attitudes (CA) among the sample of non-professional rugby players in Ireland. The RoCKAS consists of 55 questions divided into 5 sections with 2 scores: a concussion knowledge index (CKI) and concussion attitude index (CAI), totalling 40 questions. The remaining 15 questions include 12 distractor items and 3 validity questions that were not included in the overall scores. A 16 concussion item checklist forms part of the concussion knowledge index.

CKI was assessed in Sects. 1, 3, and 5 totalling 25 items. There was 17 true/false questions in Sect. 1 and 2, and 8 symptom recognition questions (with 8 non-scoring distractors) in Sect. 3. This meant a total range score of 0–25 was possible.

The CAI included 15 items from Sects. 2 and 4. A range score of 15–75 was possible as a 5 point Likert scale was used for each item. Depending on the specific question, indicating Strongly Agree or Strongly Disagree was either assigned a score of 1 or 5 respectively. The 5–item Likert scale responses (CAI) were also dichotomized into “safe” and “unsafe” responses, and neutral responses were not coded [10]. This was reported as a percentage of participants selecting a “safe” response. Aggregating items from the CKI and CAI sections generated a comprehensive RoCKAS score ranging from 15 to 100 points.

No validated outcome measure of CTE knowledge exists, so additional questions derived from current scientific literature were added, forming a six-item true/false section. Each correct answer earned 1 point, resulting in scores ranging from 0 to 6. This formed the CTE knowledge index (CTE-KI). To ensure quality, a panel of emergency medicine doctors from Cork University Hospital (CUH) reviewed and analysed the full questionnaire. A small pilot study involving 7 players from a rugby club in Cork was also done to further identify possible errors and to ensure that the questionnaire was understandable. It also ensured that data was being recorded correctly. The pilot study helped to identify that Irish Players do not use the word “Cleets”, they use the term “Rugby Boots”. None of the data gathered from the pilot study was included in the main study.

## Data analysis

Data was analysed on Statistical Package for Social Sciences (SPSS) version 29. For the survey to be considered valid and included in the analysis, participants were required to provide correct answers for at least two out of three validity scale questions and complete a minimum of 90% of the questions in both the CKI or CAI section [14].

The lead author of a previous concussion study [9] granted permission for this study to present its data in a similar format. The findings were presented by categorising players into those who play rugby at a club level and those who play rugby at a school level. Measures of central tendency (Mean) and measures of variance (Standard deviation) were utilized to evaluate the knowledge and attitudes of the respondents. The homogeneity of variance and normality within the data were examined using Levene’s test and Kolmogorov–Smirnov test. The dataset exhibited an approximately normal distribution. Independent t-tests and ANOVA tests examined the impact demographic variables had on CKI, CAI and CTE-KI. The Welsch test was used for unequal sample sizes and heterogeneity of variances. Alpha level was set at 0.05. Pearson correlation coefficient was used to test for a correlation between CKI, CAI and CTE-KI. The Chi-square test was used to analyse the difference in percentages of agreement between club and school players in relation to statements in the CKI, CAI and CTE-KI.

## Results

A total of 537 players (87.2% male, 12.8% female) participated in the study. Out of the 537 participants, 69.8% (n = 375) were club players, and 30.2% (n = 162) were school players. Two players were removed due to failure to pass the validity scale. Six players were excluded due to incomplete questionnaires, resulting in 529 players (clubs = 367, schools = 162) being included in the study. A total of 60.3% (n = 319, clubs = 276, schools = 43) completed the survey in person. A total of 39.7% (n = 210, clubs = 91, schools = 119) completed the survey online. Table 1 shows the demographic statistics.

**Table 1:** Player Demographics

	Players	
	Clubs (*n* = 367)	Schools (*n* = 162)
Demographics	Total (%)	Total (%)
Gender		
Male	299 (81.5)	162 (100)
Female	68 (18.5)	0
Age		
16–17	25 (6.8)	128 (79)
18–24	215 (58.6)	34 (21)
25–34	110 (29.9)	0
> 35	17 (4.6)	0
Club/School location		
Leinster	147 (40.1)	125 (77.2)
Munster	108 (29.4)	20 (12.3)
Ulster	82 (22.3)	17 (10.1)
Connacht	30 (8.2)	0
Years Playing experience		
0–1	8 (2.2)	1 (0.6)
1–2	20 (5.4)	2 (1.2)
3–5	35 (9.5)	32 (19.8)
6–10	89 (24.3)	74 (45.7)
11–15	87 (23.7)	52 (32.1)
> 15	128 (34.8)	1 (0.6)
Playing Position		
Front Row	87 (23.7)	34 (20.9)
Lock	51 (13.9)	14 (8.6)
Loose Forward	73 (19.9)	32 (19.8)
Scrumhalf	33 (8.9)	15 (9.3)
Outhalf	20 (5.4)	14 (8.6)
Centre	41 (11.1)	13 (8.0)
Back-Three	60 (16.3)	40 (24.7)
Suspected Previous concussion		
0	94 (25.6)	49 (30.2)
1–2	129 (35.1)	75 (46.3)
3–5	109 (29.7)	36 (22.2)
6–10	32 (8.7)	1 (0.6)
> 10	3 (0.8)	1 (0.6)
Medically Diagnosed concussion		
0	200 (54.5)	96 (59.2)
1–2	109 (29.7)	59 (36.4)
3–5	50 (13.6)	7 (4.3)
6–10	8 (2.2)	0 (0)

## Previous concussion education

Of the total participants, 87.5% indicated that they had received previous concussion education. The top sources were healthcare professionals (56.9%), IRFU concussion card [4] (54.4%), coaches (54.3%), and web resources (33.8%).

## RoCKAS

The club and school players achieved mean scores of 85.2 ± 8.0 (range 58–99) and 80.1 ± 7.6 (range 58–98) respectively.

## Correlation between CKI, CAI, and CTE-KI

Results showed there is a weak, positive correlation between CKI and CAI (r = 0.28, p < 0.001). Additionally, there was a weak positive correlation between CKI and CTE-KI (r = 0.24, p < 0.001), as well as between CAI and CTE-KI (r = 0.19, p < 0.001) respectively.

## Concussion knowledge

The mean CKI scores for the club and school players were 20.3 ± 2.3 (range 12–25) and 19.8 ± 2.6 (range 10–25) respectively. Club players scored significantly higher than schools players on CKI (M = 20.3 vs 19.8, t_527=_2.18, p = 0.03). Table 2 shows the percentage of players who correctly identified the signs and symptoms of concussion.

**Table 2:** Player’s knowledge of concussion symptoms

		% Correct	
		Clubs (*n* = 367)	Schools (*n* = 162)
	Symptom		
	Headache	98.9	99.4
	Sensitivity to Light	91.8	81.5
	Difficulty Remembering	94.0	87.0
	Drowsiness	79.8	72.2
	Feeling in a “Fog”	86.1	64.5
	Feeling Slowed Down	77.7	74.7
	Difficulty Concentrating	93.7	89.5
	Dizziness	97.8	96.9

Table 3 shows the percentage of club and school players who correctly identified CK statements. Common misconceptions for both player groups included statements relating to: experiencing coma while being knocked out (correct response: clubs: 14.7%, schools: 10.5%), structural damage *typically* being found on brain imaging (correct response: clubs: 30.8%, schools: 20.2%), and the effects of concussion on cognitive function (correct response: clubs: 25.9%, schools: 37%).

**Table 3:** Statements and scenarios evaluating the Concussion Knowledge Index (CKI) of the player and coach groups

Statements		% Correct	
		Clubs (*n* = 367)	Schools (*n* = 162)
	True Statements		
1	There is a possible risk of death if a second concussion occurs before the first one has healed	91.0	83.9
2	People who have had one concussion are more likely to have another concussion	70.6	53.7
3	Symptoms of a concussion can last for several weeks	97.0	91.2
4	After 10 days, symptoms of a concussion are usually completely gone	48.2	53.1
5	Concussions can sometimes lead to emotional disruptions	92.9	82.1
6	An athlete who gets knocked out after getting a concussion is experiencing a coma	14.7	10.5
	False Statements		
7	In order to be diagnosed with a concussion, you have to be knocked out	95.6	98.8
8	A concussion can only occur if there is a direct hit to the head	81.7	77.8
9	Being knocked unconscious always causes permanent damage to the brain	70.0	80.1
10	Sometimes a second concussion can help a person remember things that were forgotten after a first concussion	89.1	91.3
11	After a concussion occurs, brain imaging (e.g., CAT Scan, MRI, X-Ray, etc.) typically shows visible physical damage (e.g., bruise, blood clot) to the brain	30.8	20.2
12	If you receive one concussion and you have never had a concussion before, you will become less intelligent	91.3	96.3
13	After a concussion, people can forget who they are and not recognize others but be perfect in every other way	25.9	37.0
14	There is rarely a risk to long-term health and well-being from multiple concussions	81.4	75.3
	Scenarios		
15	A single concussion will affect a player’s health and well-being negatively	68.9	86.4
16	A history of multiple concussions will affect a player’s health and well-being negatively	85.6	87.0
17	Playing with concussion symptoms will not affect a player’s performance	94.8	90.1

## Concussion attitude

The mean CAI scores for club and school players were 64.9 ± 7.0 (range 39–75) and 60.4 ± 6.7 (range 29–75) respectively. Club players scored significantly higher than school players on CAI (M = 64.9 vs 60.4 t_527_ = 6.8, p =  < 0.001). Table 4 shows the percentage of club and school players who correctly identified CA statements. The 5–item Likert scale responses (CAI) were dichotomized into “safe” and “unsafe” responses. Neutral responses were not coded. Participants from clubs had a mean safe response of 85.7% compared to 77% for school participants.

**Table 4:** Statements and scenarios evaluating the Concussion Attitudes Index (CAI) of the club and school player groups

Statements					
		Clubs (*n* = 367)		Schools (*n* = 162)	
	Statements	% safe	% unsafe	%safe	%unsafe
1	I would continue playing a sport while also having a headache that resulted from a minor concussion	69.5	14.2	41.9	35.2
2	I feel that coaches need to be extremely cautious when determining whether an athlete should return to play	96.5	1.6	90.1	1.1
3	I feel that concussions are less important than other injuries	90.1	2.7	80.2	7.4
4	I feel that an athlete has a responsibility to return to a game even if it means playing while still experiencing symptoms of a concussion	88.8	6.3	79.6	6.2
5	I feel that an athlete who is knocked unconscious should be taken to the emergency room	76.0	10.1	72.8	11.7
	Scenarios				
6	I feel that the coach made the right decision to keep a fellow concussed teammate off the field, even though we lost the game	93.5	11.7	94.4	3.7
7	My teammates would feel that the coach made the right decision to keep a fellow concussed teammate off the field, even though we lost the game	83.9	7.4	74.7	11.7
8	I feel that a concussed player should have returned to play during the first game of the season	92.4	2.5	91.4	1.2
9	Most players would feel that a concussed player should have returned to play during the first game of the season	85.8	5.7	74.1	6.2
10	I feel that a concussed player should have returned to play during the semi-final playoff game	88.8	4.1	75.6	10.5
11	Most athletes feel that a concussed player should have returned to play during the semi-final playoff game	77.9	8.9	61.1	19.8
12	I feel that the physiotherapist, rather than the player, should make the decision about a player returning to play	85.5	6.5	85.9	7.4
13	Most players would feel that the physiotherapist rather than the player should make the decision about returning a player to play	81.5	8.4	79.6	6.2
14	I feel that a player with concussion symptoms should tell the coach about the symptoms even if its two hours before the game	94.1	1.9	85.1	2.5
15	Most athletes would feel that a player with concussion symptoms should tell the coach about the symptoms even if it is two hours before the game	81.1	7.6	68.5	9.9

Table 4 illustrates variances in individual attitudes ("I feel…") and perceived attitudes ("Most players…") when examining a hypothetical scenario involving the first game of the season versus a semi-final game, as reflected in statements 8–11. Both club and school players had a lower percentage of safe responses for each scenario.

## Chronic traumatic encephalopathy knowledge

The mean CTE-KI for club and school players were 5.0 ± 0.9 (range 1–6) and 4.8 ± 1.1 (range 0–6) respectively.

Club players scored significantly higher than school players on CTE-KI (M = 5.0 vs 4.8 t_273=_ 2.1, p = 0.034). Table 5 shows the percentage of players who correctly answered the statements relating to CTE. The statements with the least amount of correct responses included “symptoms of CTE can be witnessed immediately following a concussion” (correct response: clubs: 58.3%, schools: 58.1%), meaning 41.7% of club players and 41.9% of school players responded incorrectly, and “Wearing a scrum cap can prevent you from getting CTE” (correct response: Clubs: 68.4%, schools: 56.2%), meaning 32.6% of club players and 33.8% of school players responded incorrectly.

**Table 5:** Player’s knowledge of Chronic Traumatic Encephalopathy

		% Correct	
		Clubs (*n* = 367)	Schools (*n* = 162)
	Statement		
	*True statements* Symptoms of CTE include memory loss, confusion, and impaired judgement	97.0	90.1
	CTE is caused by repetitive blows to the head *False Statements*	86.9	89.5
	Only professional athletes are diagnosed with CTE	97.5	93.2
	Wearing a scrum cap can prevent you from getting CTE	68.4	56.2
	Symptoms of CTE can be witnessed immediately following a concussion	58.3	58.1
	You need to be knocked out (lose consciousness) to suffer from CTE	88.6	88.9

## Demographic variable analysis

The player outcome measures (CKI, CAI, and CTE-KI) were analysed by the demographic variables (Table 6).

**Table 6:** Player concussion knowledge, concussion attitude and Chronic Traumatic Encephalopathy knowledge scores evaluated by gender, years playing experience, and number of concussions

	Players (Clubs & Schools)														
	CKI					CAI				CTE-KI					
	*Valid N*	*Mean*	*SD*	*95% CI*	*p Value*	*Mean*	*SD*	*95% CI*	*p Value*	*Mean*			*SD*	*95% CI*	*p Value*
Gender															
Male	461	20.0	2.5	[19.8 20.3]	0.064	62.9^a^	7.3	[62.3 63.4]	< 0.001	4.9			1.1	[4.8 5.0]	0.739
Female	68	20.6	2.1	[20.1 21.1]		67.3^a^	5.4	[65.9 68.6]		4.9			0.9	[4.7 5.2]	
Level															
Club	367	20.3^b^	2.3	[20.0 20.5]	0.037	64.9^b^	7.0	[64.2 65.6]	< 0.001	5.0			0.9	[4.9 5.1]	0.034
School	162	19.8^b^	2.6	[19.4 20.2]		60.4^b^	6.7	[59.3 61.4]		4.8			1.1	[4.6 4.9]	
Survey Response															
Online	210	20.0	2.6	[19.7 20.4]	0.657	61.3 ^c^	6.7	[60.4 62.2]	< 0.001	4.9			1.0	[4.8 5.1]	0.844
In-Person	319	20.1	2.3	[19.9 20.4]		65.0 ^c^	7.2	[64.2 65.8]		4.9			1.0	[4.8 5.0]	
Years Playing Experience															
0–1	9	19.3	1.2	[18.4 20.3]	0.157	66.6	4.4	[63.2 69.9]	0.074	5.2			1.0	[4.5 6.0]	0.079
1–2	22	20.2	2.5	[19.1 21.3]		66.3	5.7	[63.8 68.8]		4.5			1.1	[4.0 4.9]	
3–5	67	20.3	2.4	[19.8 20.9]		61.7	8.2	[59.7 63.7]		4.8			1.1	[4.6 5.1]	
6–10	163	19.7	2.7	[19.3 20.1]		62.2	7.3	[61.1 63.3]		4.8			1.1	[4.7 5.0]	
11–15	139	20.2	2.2	[19.8 20.6]		63.4	6.9	[62.6 64.8]		5.0			0.9	[4.9 5.2]	
> 15	129	20.4	2.2	[20.1 20.8]		65.3	6.6	[64.2 66.5]		5.0			1.0	[4.8 5.1]	
0–10	261	19.9	2.6	[19.6 20.2]	0.066	62.6^d^	7.5	[61.7 63.5]	0.030	4.8^d^			1.1	[4.7 4.9]	0.024
≥ 11	268	20.3	2.3	[20.1 20.6]		64.5^d^	6.8	[63.6 65.3]		5.0^d^			0.9	[4.9 5.1]	
Previous Medically Diagnosed Concussion History															
0	296	19.9	2.5	[19.6 20.2]	0.140	63.1	7.3	[62.2 63.9]	0.094	4.8			1.1	[4.7 4.9]	0.058
1–2	168	20.2	2.5	[19.9 20.6]		63.5	7.1	[62.5 64.6]		5.1			0.9	[4.9 5.2]	
3–5	57	20.6	1.8	[20.1 21.1]		65.6	7.0	[63.7 67.4]		4.9			0.9	[4.6 5.1]	
6–10	8	21.0	1.6	[19.7 22.3]		65.7	5.6	[61.1 70.4]		5.0			0.5	[4.6 5.4]	
Male Previous Medically Diagnosed Concussion History															
0–2	402	20.0	2.6	[19.7 20.2]	0.071	62.6^e^	7.2	[19.7 20.2]	0.007	4.9			1.0	[4.8 5.0]	0.581
≥ 3	59	20.4	1.7	[20.1 21.1]		65.5^e^	6.1	[20.1 21.1]		4.8			0.9	[4.6 5.1]	
Female Previous Medically Diagnosed Concussion History															
0–2	62	20.4	2.1	[19.9 20.9]	0.182	67.4	5.6	[66.1 68.9]	0.406	4.9			0.9	[4.6 5.1]	0.130
≥ 3	6	22.5	1.0	[21.4 23.6]		65.5	2.6	[62.8 68.2]		5.5			0.5	[4.9 6.1]	

As indicated, significant differences were found between the following groups

^a^ Males and Females

^b^ Clubs and Schools

^c^ Online and In-Person Survey

^d^ All Players 0–10 and ≥ 11 Years Playing Experience

^e^ Males ≥ 3 and 0–2 Previous Medically Diagnosed Concussions

No significant differences for CKI (M = 20.0 vs 20.6, p = 0.064) and CTE-KI (M = 4.9 vs 4.9, p = 0.739) were found between male and female players. Females score significantly higher than males on CAI (M = 67.3 vs 62.9, t_107_ = 5.8, p =  < 0.001).

No significant differences were observed between the groups by years playing experience. However, when combining groups into 0–10 years and ≥ 11 years playing experience, it was shown that players with ≥ 11 years playing experience scored significantly higher on CAI (M = 64.5 vs 62.6, t_527_ = 3.0, p = 0.03) and CTE-KI (M = 5.0 vs 4.8, t_527_ = 2.3, p = 0.024). Players with ≥ 11 years playing experience also scored higher on CKI (M = 20.3 vs 19.9). However, these differences were not significant (p = 0.066).

Male and Female players with more previous concussions tended to score higher on the CKI and CAI than those with less previous concussions, but the results were not significant (Table 6). Males CAI improved with the number of previous concussions, and a significant improvement in males CAI was observed between males with ≥ 3 and 0–2 concussions (M = 65.5 vs 62.6, t_452_ = 2.7, p = 0.007). While the reverse trend was observed in females for CAI, these differences were not significant. Previous concussion history by gender did not show any significant differences for CTE-KI.

The impact that the remaining variables had on the outcome measures are outlined in Table 6.

## Discussion

The purpose of this study was to examine current concussion and chronic traumatic encephalopathy related knowledge and attitudes in a sample of non-professional rugby players. This study sample is large relative to other studies done in Ireland [9, 10], and elsewhere [12], but similar in sample size to others [11].

## Concussion knowledge

Compared to previous research with New Zealand (NZ) high school players [11] and South African (SA) amateur players [12], CKI levels in the current sample were higher (20.1 ± 2.5 *vs* 18.6 ± 2.4 *vs* 18.8 ± 2.4). Players in Ireland were able to identify concussion signs and symptoms correctly 78% of the time. Comparatively, NZ high school players scored higher in the concussion symptom subsection of CKI (86%)^10^, while SA junior and senior amateur players scored lower (66% & 63%)^11^ using the same RoCKAS evaluation methods."Headaches"and"dizziness"remain the most commonly identified symptoms [8–12], while"feeling slowed down,""feeling in a fog,"and"drowsiness"are the least recognised, consistent with other studies^5−9^.

O’Connell et al*.,* (2016) [8] assessed non-professional Irish rugby players knowledge and attitudes without using.

the RoCKAS survey. Their study found that 42.6% of participants falsely believed you must be hit on the head to be concussed, whereas only 18.3% of club and 22.2% of school players believed this to be true in this study. Additionally, 63.2% of players in O’Connell et al*.*’s (2016) study knew that you did not have to lose consciousness to receive a concussion, compared to 95.6% of club players and 98.8% of school players in this study. Despite the limitations of comparing data from two different measurement instruments, it is possible overtime that players concussion knowledge in Ireland has improved.

## Concussion attitudes

Club players had safer overall concussion attitudes (85.7% safe) compared to school players (77% safe). Somewhat concerning was that 35.2% of school players demonstrated unsafe attitudes towards the statement “I would continue playing a sport while also having a headache that resulted from a minor concussion”.

It is evident that some players are still influenced by important matches, as evidenced by the responses to statements 8–11 in Table 4. However, this represents an improvement from previous studies by Delahunty et al*.,* (2015) [10] and O’Connell et al*.,* (2016) [8], where 72.5% and 75% of Irish players, respectively, stated they would have continued playing in an important match even if suffering from a concussion.

## Chronic traumatic encephalopathy

As far as this study’s authors are aware, this is the first study to assess rugby players’ knowledge of CTE. Only 8.3% of participants stated that they had received prior CTE information, and 76.3% were'not familiar at all'or only'slightly familiar'with CTE. Despite this, symptom related knowledge of CTE was high with the vast majority of participants correctly identifying that symptoms of CTE include memory loss, confusion, and impaired judgemental abilities. However, misconceptions included the belief that symptoms appear immediately after a concussion and that scrumcaps prevent CTE. These gaps in knowledge may stem from the fact that the research surrounding CTE is relatively new, and that educational materials surrounding CTE are limited and lagging behind current research developments. Eighty-one point one percent (81.1%) of players stated that they would be in favour of receiving further information (Don’t know: 11.9%, No: 7%).

## Practical implications

The study results suggest a positive shift in Irish player attitudes towards a safer approach overall, which may reflect the IRFU’s ongoing efforts to promote safer rugby practices at both club and school levels [30]. This study recommends that renewed educational efforts should highlight that the specific circumstances and perceived importance of a game should not be factors that influence a player’s decision making to keep playing.

Finally, participants indicated that organisations such as the IRFU and World Rugby were their preferred source of educational materials on CTE. These bodies should take a leading role in producing and disseminating clear, credible, and engaging educational materials related to CTE. This study recommends that up-to-date, evidence-based information on CTE be integrated into existing concussion education resources provided by these bodies [15, 16]. This could be through a dedicated section that reflects the most current scientific understanding of the condition. Coaches and other key personnel within the school and club environment should be supported and encouraged to proactively convey this information to players. Visiting educational resources sponsored by the IRFU during the early season or pre-season may help maximise engagement and awareness. This information could be delivered through in-person workshops, online modules, or printed materials targeting coaches and players across all levels of participation. It is possible that improvements in concussion-related behaviours may be seen with further insight and understanding into the potential long term complications of this condition.

## Limitations

A limitation of this study is the challenge in interpreting players knowledge of CTE. This research marks the initial attempt to gauge such understanding. The questions may have been too simplified. Nonetheless, the finding that 41.7% of club and 41.9% of school players wrongly believed that CTE symptoms appear immediately after a concussion, highlights a substantial gap in knowledge about fundamental aspects of the condition. Future studies should expand on these findings, delving deeper into players knowledge of and attitudes towards CTE.

A further limitation of the study is that participants might have influenced each other's answers, though efforts were made to minimise this by separating them and discouraging conversation during the survey completion. Similarly, the online survey contained a sentence asking participants to complete the survey alone. Finally, the study assumes respondents answered truthfully without societal response bias.

## Conclusion

In this study, the higher concussion knowledge and safer concussion attitudes that were demonstrated compared to previous studies in Ireland is probably a testament to the concussion educational content promoted by the IRFU, education from coaches, and education from healthcare professionals among others. Players showed good concussion knowledge, indicating other factors such as the desire to play, performance pressure, and lack of awareness of long-term risks may still contribute to unsafe attitudes. This study highlights the need for renewed efforts, particularly regarding concussion attitudes among school players, as a significant proportion of them continue to exhibit unsafe attitudes toward concussion.

The study revealed a significant knowledge gap about CTE among participants regarding symptom onset and potential preventative measures. However, an encouraging aspect of the study was the participants'genuine interest in learning about CTE, highlighting a strong willingness to engage in educational initiatives.

## Supplementary Information

Below is the link to the electronic supplementary material.

MOESM 1 (XLSX 559 KB)
